# The effects of tabletop gaming on patients with mild cognitive impairment and Alzheimer’s disease: A feasibility study

**DOI:** 10.20407/fmj.2025-037

**Published:** 2026-05-14

**Authors:** Masaki Kamiya, Aiko Osawa, Hitoshi Kagaya, Izumi Kondo

**Affiliations:** 1 Department of Rehabilitation, National Center for Geriatrics and Gerontology, Obu, Aichi, Japan; 2 Tokyo Metropolitan Institute for Geriatrics and Gerontology, Itabashi, Tokyo, Japan

**Keywords:** Mild cognitive impairment, Alzheimer’s disease, Randomized crossover design, Gaming, Brain training

## Abstract

**Objectives:**

This study examined the feasibility of patients with mild cognitive impairment (MCI) and Alzheimer’s disease (AD) performing gaming tasks.

**Methods:**

Ten patients with MCI and nine patients with AD participated. Gaming and tabletop tasks were the two types of interventions designed for this study. Following a randomized crossover design, the two interventions were conducted at a rate of 1 hour per week for 4 weeks. The Genki! Ha•tsu•ra•tsu Trepachi-table (TOYOMARU INDUSTRY CO., LTD., Aichi, Japan) device was used for the gaming intervention. Using this table-shaped gaming device with cognitive training components, one to four players can compete in various gaming challenges on a touchscreen mounted on the top of the table. For the tabletop task intervention, patients completed arithmetic and spot-the-difference exercises as cognitive training activities. A visual analog scale was used to quantify mood before and after each intervention session, and a reaction scale measuring the “autonomy” and “degree of participation” of patients was completed after each intervention.

**Results:**

The gaming intervention significantly increased the visual analog scale score of the AD group (p=0.031). The MCI group had significantly higher autonomy reaction scale scores during the gaming intervention than the AD group (p=0.007).

**Conclusions:**

Activities using gaming technology may enhance the willingness and mood of senior citizens with diminished cognitive capabilities and could potentially support ongoing brain training.

## Introduction

The frequency of dementia is increasing in Japan, and implementing dementia countermeasures is critical.^1^ Recent studies have revealed the effectiveness of non-drug therapy in slowing the progression of dementia.^2^ In Japan, rehabilitation for patients with dementia has been shown to enhance overall cognitive performance, behavioral and psychosocial symptoms of dementia, and activities of daily living^3^; however, if the frequency of training declines, patients may revert to their pre-intervention state, making it difficult to maintain training effects. Thus, it is critical to aim for active and continuous rehabilitation in people with dementia.^4^

In addition to conventional interventions, recreational interventions using cutting-edge technology, such as robots and electronic devices, have been drawing attention. Interventions using gaming devices increased gray matter volume and improved cognitive functions, such as executive function, processing speed, and working memory.^5,6^ Positive performance feedback during gaming tasks promotes brain function and enhances motivation and adherence in older adults through the reward system.^7^ However, the results of task-based brain training were unsatisfactory in a large epidemiological study using online games, and it is vital to explore intervention strategies that can keep people motivated.^8^

An important consideration is that the neural mechanisms underlying motivation and emotional engagement differ between mild cognitive impairment (MCI) and Alzheimer’s disease (AD), and these differences are likely to directly influence the responsiveness to, and design of, task-based interventions. In individuals with MCI, reward learning and sensitivity to positive feedback are relatively preserved.^9^ Accordingly, tasks incorporating feedback are more likely to enhance motivation and engagement in this population. By contrast, individuals with AD frequently exhibit apathy and reduced task motivation; however, certain aspects of emotional processing remain relatively preserved across disease stages.^10^ Therefore, emotionally meaningful feedback may contribute to sustained participation and the maintenance of autonomy during task performance, even in AD.

These findings suggest that MCI and AD are characterized by distinct motivational and emotional response profiles, which may influence participation behavior, responsiveness, and feasibility in task-based interventions. Accordingly, it is plausible that the same tabletop gaming intervention may elicit different patterns of engagement and emotional response depending on disease stage. Examining such potential differences may provide important insights into the feasibility and acceptability of game-based interventions across the dementia spectrum.

The primary objective of this study was to evaluate the feasibility of conducting a randomized adaptive crossover trial involving tabletop gaming interventions in individuals with MCI or AD. Feasibility was assessed in terms of recruitment and retention rates, intervention adherence, and the ability to collect outcome data. Given previous findings on preserved reward sensitivity in MCI and relatively preserved emotional processing in AD, we hypothesized that game tasks would promote positive emotions and be feasible in both groups. In addition, we explored whether emotional responses in the AD group might be comparable to those observed in individuals with MCI despite greater cognitive decline.

## Methods

### Study participants

The study included 24 individuals with MCI (n=13) or AD (n=12) who attended our outpatient rehabilitation center between April 2021 and April 2022. Participants were recruited from routine rehabilitation classes conducted at our center. The attending therapists explained the study procedures both verbally and in writing, and informed consent was obtained from each participant and their family members before enrollment. As this feasibility study was not designed to verify effectiveness, no prior sample size calculation was performed. Therefore, all participants available during the study period were included. All patients with MCI had amnestic MCI according to Petersen’s criteria, and all patients with AD had a diagnosis of probable or possible AD according to criteria published by the National Institute of Neurological and Communicative Disorders and Stroke and the Alzheimer’s Disease and Related Disorders Association. Patients who exhibited dementia other than MCI or AD, and those incapable of comprehending the intervention, were excluded.

### Procedure

**Figure 1:**
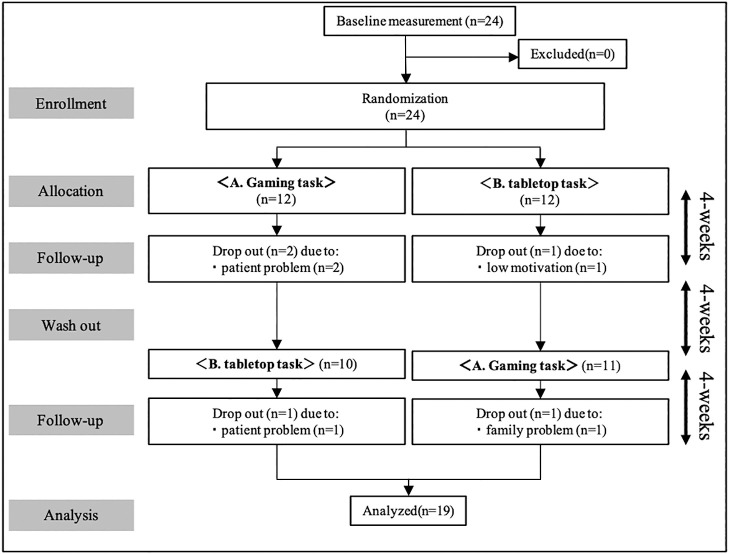
Flowchart of participants in study. A randomized crossover design.

This was an exploratory, randomized, adaptive crossover trial (Figure 1). Gaming and tabletop tasks were the two types of interventions designed in this study. Each intervention was conducted once per week for 1 hour over 4 consecutive weeks. After a 4-week washout period, the participants crossed over to other interventions following the same schedule. Thus, all participants completed eight sessions (four sessions of gaming tasks and four sessions of tabletop tasks), with the order of interventions counterbalanced between groups. The washout period was determined according to a crossover study by Han et al. (2017).^11^

The device used for the gaming intervention was the Genki! Ha•tsu•ra•tsu Trepachi-table (TOYOMARU INDUSTRY CO., LTD., Aichi, Japan) (Supplemental Figure 1). Unlike conventional video games, this device has a monitor embedded in the tabletop, allowing older adults to collaboratively engage around the table. Additionally, it does not require complex controllers and allows for intuitive operation via a touch panel, making it accessible and easy to use for older adults with cognitive impairment. In this study, the gaming tasks consisted of seven games designed to facilitate understanding even in participants with relatively impaired cognitive function (Supplemental Figure 2). The first task, “Fruit Gathering,” required participants to find the specified fruits as quickly as possible and tap the screen repeatedly on doing so. This task was assumed to primarily require simple attentional function. The second task, “King of Maths,” involved solving as many single-digit addition and subtraction problems as possible. This task was assumed to primarily require simple attentional function. The third task, “Find the Words,” required participants to rearrange random letters to form the name of the presented picture. This task was assumed to engage language function. The fourth task, “Which is Older?,” asked participants to quickly select the older of two presented photographs. This task was assumed to require remote memory. The fifth task, “Touch Money,” involved selecting and adding randomly arranged coins and bills to reach a target amount. This task was assumed to require calculation ability and attentional function. The sixth task, “Looking for Mistakes,” required participants to identify differences between two pictures. This task was assumed to require visual search and sustained attention. Finally, the seventh task, “Which Has Changed?,” asked the participants to memorize a set of playing cards and identify which card had changed when the set was presented again. All tasks were conducted in a group setting and assumed to require memory function, which is commonly impaired in individuals with MCI and dementia.

Three tasks were conducted in each session; however, the order was not fixed (week 1: Fruit Gathering, King of Maths, and Find the Words; week 2: Fruit Gathering, Which Is Older?, and Which Has Changed?; week 3: King of Maths, Find the Words, and Touch Money; week 4: Find the Words, Which Is Older?, and Looking for Mistakes). Each task, including a brief explanation and practice, took approximately 20 minutes, and the total session time was approximately 1 hour.

Because the participants all had cognitive impairment, we selected tasks that could be completed within the allotted session time. Task selection was determined through prior discussion among the researchers, during which the following principles were established. First, each session was required to include at least one low-difficulty, easy-to-introduce task, either Fruit Gathering or Find the Words, to facilitate participant engagement and ensure a smooth transition into each session. Second, the remaining two tasks were selected to stimulate cognitive domains other than attention or language, thereby targeting different cognitive functions.

In particular, tasks assumed to require the simultaneous engagement of multiple cognitive functions, and to impose relatively higher cognitive demands, were intentionally incorporated. This approach was designed to ensure that each session systematically covered a broad range of cognitive domains. Accordingly, task selection for each session was conducted in a structured manner, including one introductory task in combination with two tasks targeting different cognitive domains.

The tabletop task, established as the control condition, was designed to correspond to gaming tasks. Following the attention process training principles of Sohlberg and Mateer,^12^ peripheral, calculation, word recall, spot-the-difference, and picture card memory tasks were developed and used. These tasks were conducted individually, and no fixed task composition was predetermined for each session.

All sessions were facilitated by a research assistant who was not a co-investigator in this study and had no specialized knowledge of dementia. To avoid increased cognitive load or competition effects, scores were not displayed to the participants, and only positive feedback was provided.

### Outcome measures

This study aimed to evaluate the emotional and behavioral feasibility of the intervention; therefore, indicators focusing on participants’ engagement, motivation, and mood were selected rather than indicators of cognitive function or physiological changes. A visual analog scale (VAS; a 10 cm line where 0 cm=“boring” and 10 cm=“fun”) was used as the primary endpoint to quantify mood before and after each intervention session. The VAS was used as the primary outcome to capture participants’ subjective enjoyment and emotional response to the intervention.^13^ Assessments using the VAS were conducted collectively for all participants before the intervention, and all participants completed the VAS assessments simultaneously after the intervention (once they had all completed their tasks).

“Reaction” was evaluated after each intervention session using a reaction scale, serving as the secondary endpoint.^14^ The scale measured the “autonomy” and “degree of participation” of patients during an activity. Autonomy was assessed as follows: 4: “Patient proceeds with the activity based on own initiative;” 3: “Patient starts the activity with encouragement;” 2: “Patient reacts to encouragement, but does not participate;” 1: “Patient does not react to any encouragement.” The degree of participation was assessed as follows: 4: “Patient engages in activity with concentration and enthusiasm;” 3: “Patient is distracted, but finishes the activity with encouragement;” 2: “Patient initially attempts activity and participates if encouraged, but gives up half way through;” and 1: “Patient does not participate at all and requires full assistance.” The reaction scale was administered by a research assistant who was not a co-investigator to ensure that the assessment results were not influenced by the intervention staff or other researchers.

### Randomization

Participants were allocated to intervention-first and control-first intervention orders using a table of random numbers. A total of 24 participants were enrolled, with 12 assigned to each order. Owing to differences in clinic days and time slots, participants with MCI and AD were not evenly distributed within each order. The study used a randomized crossover design and was conducted as an open-label trial; therefore, the participants, caregivers, and outcome assessors were not blinded to the group assignments.

### Statistical analysis

The individuals were divided into MCI and AD groups because previous studies have suggested that the nature of task engagement, including emotional and motivational responses, varies with the severity of cognitive decline.^15^ Paired t-tests were used to compare the differences in VAS scores before and after the two interventions. To evaluate possible carryover effects in this crossover study, pre- to post-intervention difference scores were compared between the gaming-task-first and gaming-task-second groups using an independent t-test. Additionally, the Mann–Whitney U test was performed to examine how the median reaction scale scores varied for each task. With a significance threshold of 5%, the data were analyzed using SPSS version 28.0 (IBM Corporation, Armonk, NY, USA).

### Ethical considerations

This study received approval from the ethics committee of the authors’ institute.

## Results

### Course of intervention and completion rate

The course of this randomized crossover study is shown in Figure 1. No participants met the study exclusion criteria. At the follow-up, two individuals assigned to the gaming task intervention dropped out for personal reasons, and another for family reasons. One individual dropped out of the tabletop task intervention owing to decreasing motivation and interest, and another for personal reasons. Therefore, the final analysis included 19 patients, for a completion rate of 79.2%.

No adverse events were reported in the intervention or control periods. The 19 participants who completed both interventions (9 in the gaming task and 10 in the tabletop task) were included in the final analysis. As participants who dropped out were excluded, a per-protocol analysis was adopted. No modifications were made to the study period. Although the trial was designed considering the possibility of adaptation, the prespecified criteria for modification were not met.

### Participants’ clinical characteristics

The clinical characteristics of the 19 analyzed participants are shown in Table 1. Ten patients with MCI [mean age, 77±7 years; median (interquartile range) Mini-Mental State Examination-Japanese (MMSE-J^16^) score, 25 (23–25)] and nine patients with AD [mean age, 79±7 years; median MMSE-J score, 19 (16–21)] were included in the analysis. There were no differences in age, gender, years of education, or other characteristics between the MCI and AD groups. The MMSE-J score was significantly lower in the AD group than in the MCI group (p<0.001).

### Comparison of pre- and post-intervention VAS scores of the MCI and AD groups (Figure 2)

After the gaming task intervention, the VAS score of the AD group increased significantly (pre-intervention, 7.1±2.1; after 4 weeks, 9.2±0.9) (p=0.031). There were no discernible changes in the MCI group. The VAS score did not differ significantly between the MCI and AD groups before and after the tabletop task intervention. In this exploratory analysis, the difference scores in the gaming-task-first and gaming-task-second groups were 0.7±1.8 and 1.8±2.0, respectively, with no significant difference (p=0.860).

### Comparison of autonomy and degree of participation (Table 2)

The MCI group had significantly higher autonomy reaction scale scores during the gaming intervention for the Touch Money, Looking for Mistakes, and Which has Changed? tasks than the AD group (p=0.015, p=0.002, and p=0.005, respectively). However, there were no significant group differences in the Fruit Gathering, King of Maths, Find the Words, and Which is Older? scores. In terms of degree of participation, there was a group difference only for the Which has Changed? task; the score was significantly higher for the MCI group.

## Discussion

### Consistency between hypotheses and results

We hypothesized that gaming tasks would promote positive emotions and would be feasible for both the MCI and AD groups, with the possibility that patients with AD might show emotional responses comparable to those of patients with MCI. The results partially supported our hypotheses. Both groups exhibited positive emotional responses; however, the AD group demonstrated lower levels of autonomy and engagement than the MCI group, suggesting that cognitive decline may have affected their participation behaviors.

### Effects of task characteristics and cognitive function on feasibility

According to the reaction scale results, participants in the MCI group showed higher levels of autonomy and engagement during the gaming intervention. In addition, for game tasks that required attention, math, remote memory, and recall, autonomy and degree of participation scores were higher, enjoyment was considerably enhanced, and completion rates were higher in the AD group. Prior research has also found that when program difficulty is adequate, group rehearsals provide a platform for peer support and improve attendance.^17,18^ According to these findings, we concluded that a tabletop gaming intervention involving numerous MCI and AD individuals is viable.

The gaming tasks included Touch Money, which requires simultaneous calculation and search; Looking for Mistakes, which requires careful visual exploration; and Which Have Changed?, involving visuospatial short-term memory. Tasks with a dual element,^19^ error-detection tasks,^20^ and visuospatial short-term memory tasks^21^ are particularly challenging for patients with AD. Accordingly, our AD group was less independent in its responses and required more prompts than the MCI group. Apathy, characterized by flat emotions, is the most common behavioral and psychological symptom of AD.^22,23^ Therefore, in AD rehabilitation, tasks of sufficient difficulty must be provided to generate motivation. Furthermore, there have been reports of older adults having difficulty using touch panels owing to vision impairments and a lack of accuracy.^24^ Our patients with AD may have had difficulty in operating the device because of visuospatial cognitive impairment. To maintain engagement with tabletop gaming training in patients with AD, it must be possible for them to accomplish tasks using their remaining functions, such as memories from the past. Tasks must be simple so that these patients can concentrate on them independently, and devices must be easy to operate. When task difficulty is appropriate for the patient, brain activity increases,^25^ implying that attention should be paid not only to the type of task but also to its difficulty. Task difficulty and cognitive demand may have influenced our results, and these factors should be considered when interpreting the between-group comparisons. The intervention was conducted once a week. A systematic review of gaming tasks aimed at cognitive enhancement in older adults with cognitive impairment reported that most studies implemented interventions at least twice a week. Thus, the task frequency in this study may have been insufficient. Future studies should consider the optimal frequency when evaluating intervention effectiveness.^26^

According to the above findings, gaming tasks were expected to promote positive emotions in both our MCI and AD groups. However, autonomy and task engagement were slightly reduced in the AD group, suggesting an impact on cognitive decline. These results imply that cognitive impairment may influence the effectiveness of interventions and should be considered in future studies.

### Gaming intervention outcomes for the MCI and AD groups

From a feasibility perspective, analyzing patients with MCI and AD as distinct groups allowed us to identify stage-specific challenges to, and facilitators of, engagement that would have been obscured had the patients been treated as a single, heterogeneous population. This distinction is particularly relevant for the design of future interventions, as it highlights the need to tailor task characteristics, feedback style, and levels of support according to cognitive and motivational capacity.

As we planned a feasibility study, we focused on the mood and reactions of participants before, during, and after the intervention. Statistically significant differences were seen in the scores on the reaction scale, which evaluated autonomy, and in the scores on the VAS, which evaluated mood.

According to reviews of intervention studies that used computers and tablets for patients with MCI or dementia, although the patients had a high degree of satisfaction with such interventions, and showed an improvement in behavioral/psychological symptoms, there was no evidence of cognitive function improvement.^27,28^ However, Hung et al. reported that an intervention for patients with dementia using tablets (iPad; Apple Corporation, Cupertino, CA, USA) gave them a sense of security.^29^ Therefore, it is desirable to investigate the effect of gaming interventions on mood and motivation in patients with MCI or AD, rather than just the effect on cognitive functions, and to use behavioral/psychological symptoms as outcome markers.

### Limitations

Because this study was exploratory and had a small number of cases, statistical significance was difficult to achieve. In future studies, the primary outcome will be the difference in cognitive function between the intervention and control groups. The sample size required for this study was calculated assuming a medium effect size (Cohen’s d=0.5–0.6), a two-sided significance level of 0.05, and a power of 0.80. A medium effect was considered reasonable because Savulich et al. (2017) reported cognitive improvement in 42 patients with MCI following a gaming-based intervention. Consequently, 44–63 participants were required per group. Considering a completion rate of 79.2%, we planned to recruit 56–80 participants per group.^30^ As we used a tabletop gaming device, future studies should investigate the effect of gaming using more familiar electronic devices, such as smartphones and personal computers.

The reaction scale used in this study exhibited a ceiling effect, and is thus insufficient to fully capture responses in individuals with MCI or AD. Future research should use more disease-specific measures, such as the Apathy Scale.^31^ Furthermore, because physiological measures (e.g., heart rate variability) and standardized behavioral or psychological scales were not used, subjective emotional changes were assessed. Therefore, the applicability of the results to other populations is limited.

In this study, gaming tasks were conducted in a group format, whereas tabletop tasks were administered individually according to the inherent characteristics of each task. Therefore, direct comparison between the two types of tasks under identical conditions was not possible, which represents a study limitation. Future research should address this limitation by standardizing the task format across conditions by, for example, implementing tabletop tasks as group-based cognitive activities or using devices that allow gaming tasks to be conducted individually. Such methodological refinements would enable more direct and rigorous comparisons between task types.

Paired t-tests and Mann–Whitney U tests were used for statistical analysis; however, these methods do not fully account for crossover and repeated-measures designs. As this was a feasibility study with a limited sample size, simple analytical methods were adopted to obtain preliminary insights. Furthermore, the crossover design may have influenced the results. Although a sufficient interval was maintained between the intervention periods and exploratory analyses suggested minimal carryover effects, an order effect could not be completely ruled out. These points should be carefully considered in future randomized controlled trials.

In conclusion, gaming device therapies are more enjoyable for patients with AD, and more amenable to voluntary participation by patients with MCI, than tabletop tasks. Therefore, activities including gaming technology may not only enhance the willingness and mood of senior citizens with diminished/impaired cognitive capabilities, but could also support ongoing brain training.

## Figures and Tables

**Figure 2:**
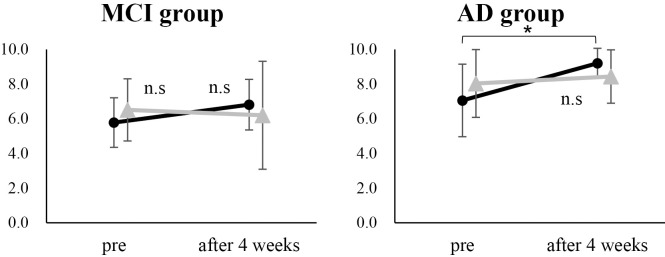
Comparison of MCI and AD’s impressions between the pre- and post-intervention. -●-Gaming task　-▲-Tabletop task Paired t-test. * Significant at the 0.05 level.

**Table 1:** Clinical characteristics of the study participants

	MCI group	AD group	P-values
N	10	9	
Age (years)^†^	77±7	79±7	0.568
Men^‡^	4 (40.0%)	4 (44.4%)	0.605
Years of education^†^	12±1	11±2	0.131
MMSE-J scores^§^	25 [23–25]	19 [16–21]	<0.001

Data are shown as mean±standard deviation or median [interquartile range]. ^†^ Unpaired t-test; ^‡^ Fisher’s exact test; ^§^ Mann–Whitney U test. MCI, mild cognitive impairment; AD, Alzheimer’s disease; MMSE-J, Mini-Mental State Examination-Japanese.

**Table 2:** Autonomy and degree of participation scores on the reaction scale

		Autonomy			Degree of participation		
Intervention	Application title	MCI group	AD group	P-values	MCI group	AD group	P-values
Gaming task	Fruit Gathering	4 [4–4]	4 [4–4]	0.289	4 [4–4]	4 [4–4]	1
Gaming task	King of Maths	4 [4–4]	4 [3–4]	0.327	4 [4–4]	4 [4–4]	1
Gaming task	Find the Words	4 [4–4]	4 [4–4]	0.611	4 [4–4]	4 [4–4]	1
Gaming task	Which is Older?	4 [4–4]	3 [3–3]	0.162	4 [4–4]	4 [4–4]	1
Gaming task	Touch Money	4 [4–4]	3 [3–4]	**0.015**	4 [4–4]	4 [4–4]	1
Gaming task	Looking for Mistakes	4 [4–4]	3 [–3]	**0.002**	4 [4–4]	4 [3–4]	0.081
Gaming task	Which has Changed?	4 [4–4]	3 [3–4]	**0.005**	4 [4–4]	3 [3–4]	**0.005**
Tabletop task	Arithmetic and spot-the-difference exercises	4 [3–4]	3 [3–4]	0.182	4 [4–4]	4 [4–4]	1

Data were analyzed using the Mann–Whitney U test and are shown as median [interquartile range].
