# Supplementary material for: The effects of tabletop gaming on patients with mild cognitive impairment and Alzheimer’s disease: A feasibility study
Source: Fujita Med J. 2026 May 14;12(3):215–21. doi: 10.20407/fmj.2025-037 (PMC13433038; doi:10.20407/fmj.2025-037)
Supplement: Supplementary file 1 — Supplemental Figures [file fmj-12-215_s1.pdf]

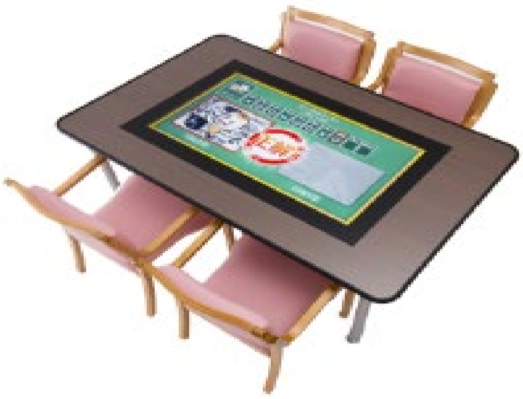

Supplemental Figure 1 **Gaming task's "Genki! Ha• tsu• ra• tsu Trepach-table" in its entirety**

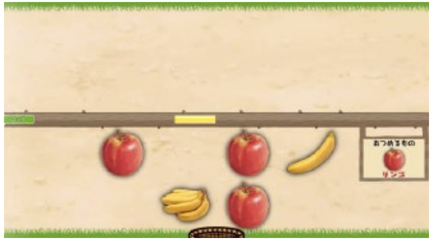

< Fruit gathering >
Simple attention task

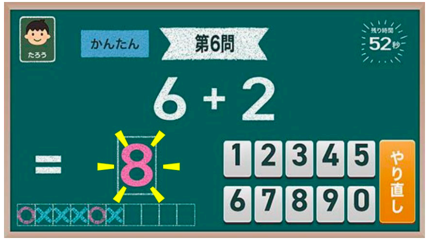

< King of maths >
Simple Calculation

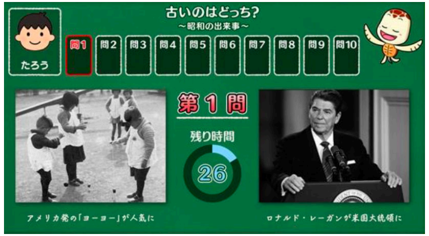

< Which is older? >
Use of long-term memory, recollection

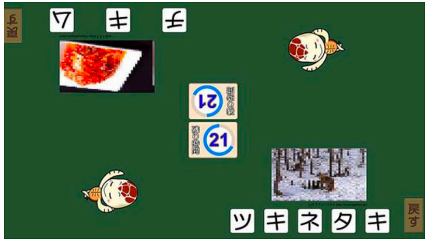

< Find the words >
Sort letters into correct words

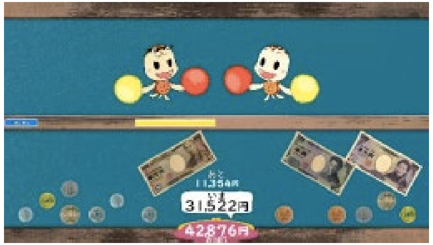

< Touch Money >
Understanding and calculating money

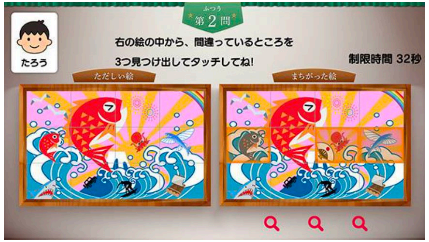

< Looking for mistakes >
Complex attention task

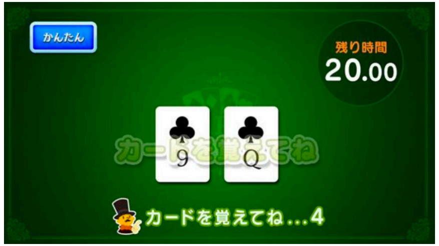

< Which has changed? >
Memory task

Supplemental Figure 2 **Gaming tasks used in this study**
